# Serial Serum Leukocyte Apoptosis Levels as Predictors of Outcome in Acute Traumatic Brain Injury

**DOI:** 10.1155/2014/720870

**Published:** 2014-04-17

**Authors:** Hung-Chen Wang, Tzu-Ming Yang, Yu-Jun Lin, Wu-Fu Chen, Jih-Tsun Ho, Yu-Tsai Lin, Aij-Lie Kwan, Cheng-Hsien Lu

**Affiliations:** ^1^Department of Neurosurgery, Kaohsiung Chang Gung Memorial Hospital and Chang Gung University College of Medicine, Kaohsiung 83301, Taiwan; ^2^Graduate Institute of Medicine, College of Medicine, Kaohsiung Medical University, Kaohsiung 80708, Taiwan; ^3^Division of Neurosurgery, Department of Surgery, Yuan's General Hospital, Kaohsiung 80249, Taiwan; ^4^Department of Biological Science, National Sun Yat-Sen University, Kaohsiung 80424, Taiwan; ^5^Department of Otolaryngology, Kaohsiung Chang Gung Memorial Hospital and Chang Gung University College of Medicine, Kaohsiung 83301, Taiwan; ^6^Departments of Neurosurgery, College of Medicine, Kaohsiung Medical University, Kaohsiung 80708, Taiwan; ^7^Department of Neurology, Kaohsiung Chang Gung Memorial Hospital and Chang Gung University College of Medicine, Kaohsiung 83301, Taiwan

## Abstract

*Background*. Apoptosis associates with secondary brain injury after traumatic brain injury (TBI). This study posits that serum leukocyte apoptosis levels in acute TBI are predictive of outcome. *Methods*. Two hundred and twenty-nine blood samples from 88 patients after acute TBI were obtained on admission and on Days 4 and 7. Serial apoptosis levels of different leukocyte subsets were examined in 88 TBI patients and 27 control subjects. *Results*. The leukocyte apoptosis was significantly higher in TBI patients than in controls. Brief unconsciousness (*P* = 0.009), motor deficits (*P* ≤ 0.001), GCS (*P* ≤ 0.001), ISS (*P* = 0.001), WBC count (*P* = 0.015), late apoptosis in lymphocytes and monocytes on Day 1 (*P* = 0.004 and *P* = 0.022, resp.), subdural hemorrhage on initial brain CT (*P* = 0.002), neurosurgical intervention (*P* ≤ 0.001), and acute posttraumatic seizure (*P* = 0.046) were significant risk factors of outcome. Only motor deficits (*P* = 0.033) and late apoptosis in monocytes on Day 1 (*P* = 0.037) were independently associated with outcome. A cutoff value of 5.72% of late apoptosis in monocytes was associated with poor outcome in acute TBI patients. *Conclusion*. There are varying degrees of apoptosis in patients following TBI and in healthy individuals. Such differential expression suggests that apoptosis in different leukocyte subsets plays an important role in outcome following injury.

## 1. Introduction

In many countries, acute traumatic brain injury (TBI) is a major cause of mortality and disability in the early decades of life [1]. An estimated 1.4 million people sustain TBI each year in the United States alone and more than 5 million people are coping with disabilities from TBI at costs of about $56 billion a year [2]. In the European epidemiology data, TBI incidence (hospitalized and fatal) is estimated to be 235 per 100,000 per year, while the case fatality rate is 11 per 100 with 775,500 new cases each year [3]. Thus, TBI is a significant health concern and an enormous socioeconomic burden.

TBI can induce oxidative stress by inflammation, ischemic-reperfusion injury, hemoglobin release, and tissue damage. Oxidative stress can further cause oxidative damage to cell components to induce cell necrosis and apoptosis [4, 5]. Traumatically induced cell death is considered to be primarily necrotic in nature [6, 7] and is characterized by swelling of the nucleus and cytoplasmic organelles, as well as by an early loss of plasma-membrane integrity and cell lysis [6]. Apoptosis, the morphologic manifestation of programmed cell death, is associated with normal central nervous system (CNS) development [8]. In contrast to necrosis, a cell undergoing apoptosis is characterized by uniform internucleosomal DNA fragmentation, nuclear shrinkage, chromatin compaction, and cytoplasmic condensation and disintegration. In addition to its role in normal physiologic cell death, apoptosis is associated with specific pathologic conditions in the CNS, including Alzheimer's and Huntington's disease [9, 10], spinal cord injury [11, 12], and cerebral ischemia [13–17]. Aside from necrotic cell death due to focal tissue damage following TBI, cell death consistent with apoptosis has been observed in the cortex, hippocampus, and thalamus in experimental brain injury [18–21].

To date, little is known about the time course of apoptotic changes in acute TBI. Much more accurate information about changes may be gained by taking serial rather than single blood samples. This prospective study aimed to evaluate the relationship between serial apoptotic changes and therapeutic outcomes in acute TBI patients.

## 2. Patients and Methods

### 2.1. Patients

This prospective study on the time course of plasma DNA levels in acute TBI patients enrolled 88 adult patients (age ≥18 years) admitted within 24 hours after onset of acute TBI to Kaohsiung Chang Gung Memorial Hospital, a 2715-bed acute-care teaching medical center in southern Taiwan providing both primary and tertiary referral care.

The diagnosis of acute TBI was confirmed by history and brain computed tomography (CT) scan. Patients were excluded if they (1) had penetrating head injury or gunshot wound; (2) were taking antiplatelet or anticoagulant drugs before the acute TBI; (3) had evidence of alcoholism or any other addictive disorders, (4) central nervous infection during hospitalization; and (5) major systemic diseases like end-stage renal disease, liver cirrhosis, or congestive heart failure. The Ethics Committee of the hospital's Institutional Review Board approved the study. All of the patients or their representatives provided written informed consent. For comparison, 27 healthy volunteers undergoing annual physical checkup were enrolled as controls.

Patients were under continuous observation and monitored at regular intervals for Glasgow Coma Scale (GCS) Score, electrocardiogram, blood pressure, pulse rate, temperature, fluid balance, and laboratory parameters. The patients were also divided into three groups according their initial GCS score: mild (GCS score 13–15), moderate (GCS score 9–12), and severe TBI (GCS score 3–8). Outcome was assessed upon discharge using the Glasgow Outcome Scale (GOS), with good outcome defined as GOS ≥4 and poor outcome as GOS ≤3. The Abbreviated Injury Score (AIS) for individual body regions was determined and the total extent of injury was calculated using the objective Injury Severity Score (ISS) upon admission [22].

All of the patients underwent brain CT scan shortly after arriving at the emergency room. Repeat brain CT scan or/and magnetic resonance imaging (MRI) was performed for any clinical deterioration (e.g., acute-onset focal neurologic deficits, seizures, status epilepticus, and progressively disturbed consciousness) and as routine postneurosurgical procedure. The principal investigator reviewed all of the available initial and follow-up CT scans and MRIs. In equivocal cases, a second observer reviewed the imaging studies. The observers/reviewers were blinded to the laboratory results at the time of clinical and radiologic assessment.

### 2.2. Blood Sampling

Two hundred and twenty-nine blood samples from 88 TBI patients and 27 blood samples from 27 control subjects were collected. Seventy-one blood samples were taken within 24 h after the onset of TBI, 81 on Day 4, and 77 on Day 7 after the onset of acute TBI, regardless of clinical deterioration. Sera were isolated from peripheral blood samples, drawn from each subject before and after the expedition. Blood samples were centrifuged at 3000 rpm for 10 min. Each serum sample was collected and frozen at −80°C prior to biochemical measurements.

### 2.3. Assessment of Leukocyte Apoptosis

#### 2.3.1. Flow Cytometry Assay for Detecting Apoptosis

*APO 2.7.* All flow cytometry assays were performed within one hour after blood extraction to ensure that the results were as close as possible to* in vivo* situation. Fixed amounts of blood were diluted 1 : 5 with PBS and 100 *μ*L was stained with 10 *μ*L of each of the following: fluorescence conjugated monoclonal antibodies against CD45-phycoerythrin (PE)-Cy5 (clone J33), CD61-fluorescein isothiocyanate (FITC; clone SZ21), and APO 2.7-PE (clone 2.7A6A3; Immunotech, Marseille, France). The blood samples were titrated at saturating concentrations. The CD45-PE-Cy5 antibody was a pan-leukocyte marker that reacted with the CD45 family of transmembrane glycoproteins, expressed on the surface of all human leukocytes. The CD61-FITC antibody was a pan-platelet marker that reacted with the GPIIb/IIIa complex (fibrinogen receptor). The APO 2.7-PE antibody reacted with a 38-kDa mitochondrial membrane protein (7A6 antigen) that was detectable on nonpermeabilized cells in the late apoptotic state [23].

*Annexin V.* Annexin V staining for early apoptosis produced similar results but was rejected for questionable reliability under fixation condition, with formaldehyde clearly biasing the staining results. Mouse immunoglobulin G-PE was a control for nonspecific staining, which did not differ from the APO2.7-PE signal on platelets, such that each subject could be used as its own control without changing the sample tube. After 30 min of incubation in the dark at room temperature, the stained samples were diluted with 0.5 mL of FACSFlow (Becton Dickinson, San Jose, CA). Flow cytometry analysis was performed immediately after staining using an Epics XL flow cytometer (Beckman Coulter, Fullerton, Calif) and CellQuest software. Five thousand CD45-PE-Cy5+ cells per sample were acquired in combined forward and side scatters and deep-red FL4 fluorescence (CD45-PE-Cy5) leukocyte gate. Another 5000 CD61-FITC+ cells per sample were acquired in combined forward and side scatters and green FL1 fluorescence (CD61-FITC) platelet gate to define a negative control threshold for measuring apoptosis, so that each subject was his/her own control.

*Annexin V-FITC 7-AAD.* Membrane phosphatidyl-serine was detected by annexin-V using a commercially available kit (Boehringer Mannheim, Indianapolis, Ind). The PBS-washed leucocytes were incubated with annexin V-FITC and 7-amino-actinomycin D (7-AAD) for 15 min at room temperature according to manufacturer's guidelines. Samples were transferred to 5 mL polypropylene tubes, diluted 900 *μ*L with Hank's balanced salt solution, and placed on ice before analysis by flow cytometry. The samples were analyzed using an Epics XL flow cytometer (Beckman Coulter, Fullerton, Calif) and CellQuest software. Fifteen thousand events were counted per sample. Low-fluorescence debris was gated out of the analysis. Leukocyte subtypes were identified according to their CD45 expression intensity and divided into neutrophils, monocytes, and lymphocytes. From hereon, white blood cells (WBC) represented total leukocytes.

Annexin V-FITC staining was identified in fluorescent-1 and 7-AAD staining in fluorescent-4. Cells were identified as follows: early apoptotic cells if they were positive for marker annexin V-FITC but negative for 7-AAD; late apoptotic cells if they were positive for annexin V-FITC and 7-AAD; dead cells if they were negative for annexin V-FITC but positive for 7-AAD; and viable cells if they were negative for annexin V-FITC and 7-AAD.

### 2.4. Data Analysis

Data were expressed as median (interquartile range [IQR]). Statistical significance was set at *P* < 0.05. Categorical variables were compared using the Chi-square test or Fisher's exact test, as appropriate, while continuous variables were assessed by the Mann-Whitney *U* test. Correlation analysis using the Spearman rank test explored the relationship between age, GCS on admission, ISS on admission, and oxidative stress levels.

Stepwise logistic regression analysis was used to evaluate the relationship between significant variables and therapeutic outcomes, with adjustments made for other potential confounding factors. Variables with zero cell count in a 2-by-2 table were eliminated from logistic analysis and only variables with strong association with poor outcome (*P* < 0.05) were included in the final model. The receiver operating characteristic (ROC) curve analysis was used to estimate an optimal cutoff value for oxidative stress levels on admission. The areas under the ROC curves (AUCs) were calculated for each parameter and compared. All of the statistical analyses were conducted using the SAS software package, version 9.1 (2002, SAS Statistical Institute, Cary, North Carolina).

## 3. Results

### 3.1. Baseline Characteristics of the Study Patients

The baseline characteristics of the 88 adult acute TBI cases and 27 controls were listed in Table 1. The acute TBI patients included 55 males (age range, 18–69 years; median age, 32 years) and 33 females (age range, 18–70 years; mean age, 46 years). By GCS score on admission, 68 (77.3%) were mild, eight (9.1%) were moderate, and 12 (13.6%) were severe TBI. The median (IQR) ISS on admission was 16 (11, 20). Eighteen patients had minor injury (ISS < 9), 19 had moderate injury (ISS 9–15), 43 had severe injury (ISS 16–24), and eight had very severe injury (ISS > 24). Eighteen (20.5%) underwent neurosurgery within 24 h after TBI, including three who had ventriculotomy, three craniotomy, four craniectomy, six craniotomy and ventriculotomy, and two craniectomy and ventriculotomy. The median (IQR) of GCS score and ISS on admission of those who received neurosurgical treatments were 7 (6, 14) and 20 (17, 25), respectively. The most common brain CT findings at presentation was traumatic SAH (45/88, 51.1%) and subdural hemorrhage (34/88, 38.6%).

### 3.2. Leukocyte Apoptosis in Patients with TBI and the Controls

The laboratory data and percentage of leukocyte apoptosis of the two groups were listed in Table 1. The WBC counts were significantly different. Except for annexin V in monocytes and annexin V+7-AAD in total leukocytes, lymphocytes, and monocytes, the percentages of apoptosis of total leukocytes and their subsets were significantly higher in the TBI patients than in the controls (Table 1).

### 3.3. Effects of Leukocyte Apoptosis on Outcome in Acute TBI Patients

Leukocyte apoptosis on presentation positively correlated with GCS score (annexin V+7-AAD in neutrophil, *P* ≤ 0.001), negatively correlated with ISS (annexin V in neutrophil and monocyte, *P* = 0.011 and 0.006, resp.), negatively correlated with WBC (APO2.7 in neutrophil and total leukocytes, *P* = 0.021 and 0.038, resp.), and negatively correlated with length of intensive care unit (ICU) stay (APO2.7 in neutrophil, annexin V in neutrophil and monocyte, *P* = 0.018, 0.026 and 0.025, resp.).

**Figure 1:**
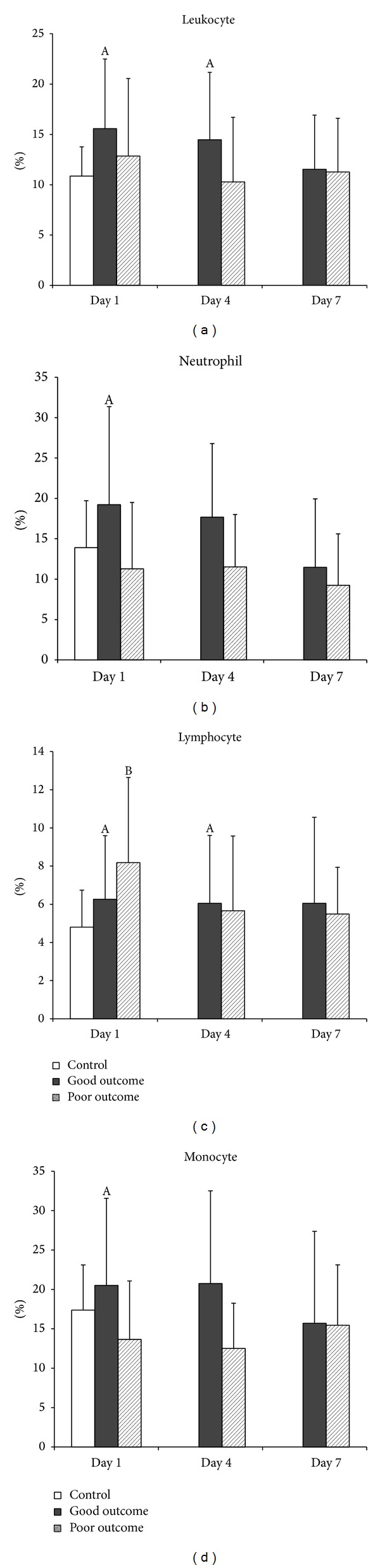
Mean percentages of early apoptosis (annexin V) in (a) total leukocytes, (b) neutrophils, (c) lymphocytes, and (d) monocytes on Days 1, 4, and 7 in patients with acute TBI and in the controls. ^A^ *P* < 0.05, acute TBI patients with good outcome versus health controls; ^B^ *P* < 0.05, acute TBI patients with poor outcome versus health controls; *P < 0.05, acute TBI patients with poor outcome versus those with good outcome, by Mann-Whitney *U* test.

**Figure 2:**
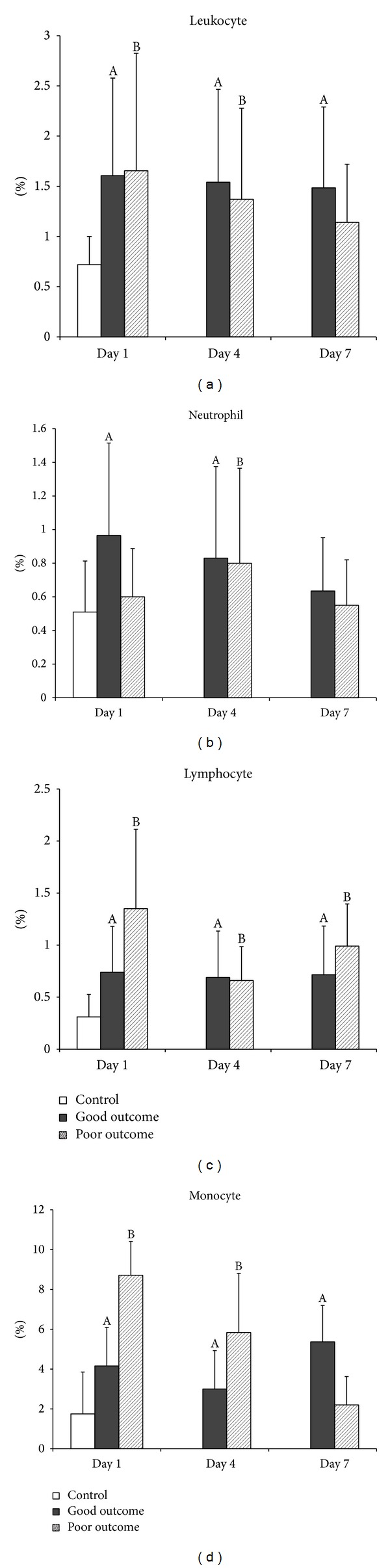
Mean percentages of mitochondrial apoptosis (APO2.7) in (a) total leukocytes, (b) neutrophils, (c) lymphocytes, and (d) monocytes on Days 1, 4, and 7 in patients with acute TBI and in the controls. ^A^ *P* < 0.05, acute TBI patients with good outcome versus health controls; ^B^ *P* < 0.05, acute TBI patients with poor outcome versus health controls; *P < 0.05, acute TBI patients with poor outcome versus those with good outcome, by Mann-Whitney *U* test.

**Figure 3:**
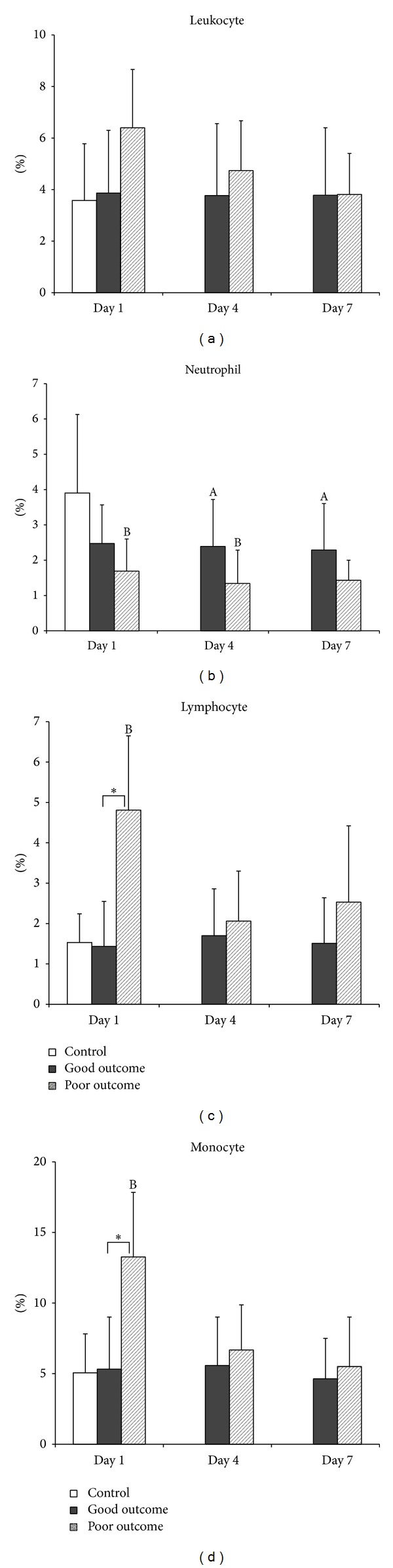
Mean percentages of late apoptosis (annexin V+7-AAD) in (a) total leukocytes, (b) neutrophils, (c) lymphocytes, and (d) monocytes on Days 1, 4, and 7 in patients with acute TBI and in the controls. ^A^ *P* < 0.05, acute TBI patients with good outcome versus health controls; ^B^ *P* < 0.05, acute TBI patients with poor outcome versus health controls; *P < 0.05, acute TBI patients with poor outcome versus those with good outcome, by Mann-Whitney *U* test.

The time course of leukocyte apoptotic changes in acute TBI patients with good and poor outcomes, as determined by GOS, was compared. The percentages of apoptosis of annexin V+7-AAD in lymphocytes and in monocytes were significantly lower in the good outcome group (median: 1.44 and 4.79, resp.) than in the poor outcome group (median: 4.81 and 11.94, resp.) on Day 1. There was statistically significant difference (*P* = 0.004 and *P* = 0.022, resp.) (Figures 3(c) and 3(d)). There was no other statistically significant difference between the two outcome groups in the percentages of apoptosis of total leukocytes and their subsets from Day 1 to Day 7 (Figures 1–3).

### 3.4. Outcome and Prognostic Factors of Acute TBI Patients

By GOS upon discharge of the 88 acute TBI patients, 77 had no disability, two had moderated disability, four had severe disability, and five became vegetative. Twelve (13.6%) had neurosurgical complications during the acute stage of TBI, including four with new onset neurologic deficits, seven with deterioration of consciousness, and nine with posttraumatic seizure (Table 2).

The clinical features, neuroimaging findings, and laboratory data of the patient groups with good (GOS ≥ 4) and poor (GOS ≤ 3) outcomes were compared. Statistical analysis revealed significant differences in brief unconsciousness on presentation (*P* = 0.009), motor deficits on presentation (*P* ≤ 0.001), GCS on presentation (*P* ≤ 0.001), ISS on presentation (*P* = 0.001), WBC count on presentation (*P* = 0.015), subdural hemorrhage on initial brain imaging (*P* = 0.002), neurosurgical intervention (*P* ≤ 0.001), acute neurosurgical complications with posttraumatic seizure (*P* = 0.046), and the percentages of apoptosis of annexin V+7-AAD in lymphocytes and in monocytes on Day 1 (*P* = 0.004 and *P* = 0.022, resp.) (Table 2).

All of these variables except for the length of ICU and hospital stay were used in the logistic regression analysis. Only motor deficits on presentation (*P* = 0.033; expectancy: 0.005; 95% CI: 0.001–0.647) and the percentages of apoptosis of annexin V+7-AAD in monocytes on Day 1 (*P* = 0.037; expectancy: 1.383; 95% CI: 1.019–1.878) were independently associated with outcome.

To determine the relationship between the percentages of apoptosis of annexin V+7-AAD in monocytes on Day 1 and outcome, the ROC curves were generated. The AUC for annexin V+7-AAD in monocytes on Day 1 was 0.748 (*P* = 0.022, 95% CI: 0.547–0.949). The cutoff value of annexin V+7-AAD in monocytes on Day 1 was 5.72% (sensitivity 75% and specificity 58.9%).

## 4. Discussion

The present study has several major findings. First, the mean percentages leukocyte apoptosis (including neutrophil, monocyte, and lymphocyte) on presentation are significantly higher in TBI patients than in healthy controls. Second, the mean percentages of late apoptosis (annexin V+7-AAD) of neutrophil on presentation positively correlate with GCS score. Third, the mean percentages of early apoptosis (annexin V) of neutrophils and monocytes on presentation negatively correlate with ISS and length of ICU stay. Fourth, the mean percentages of early apoptosis (APO2.7) of neutrophils and total leukocytes on presentation negatively correlate with WBC and length of ICU stay. Fifth, in terms of therapeutic outcomes as determined by GOS, the mean percentages of late apoptosis (annexin V+7-AAD) in lymphocytes and monocytes are significantly lower in the good outcome group (median: 1.44 and 4.79, resp.) than in the poor outcome group (median: 4.81 and 11.94, resp.) on presentation. There is a statistically significant difference (*P* = 0.004 and *P* = 0.022, resp.) (Figures 3(c) and 3(d)). Lastly, motor deficits and the mean percentages of late apoptosis (annexin V+7-AAD) in monocytes on presentation are independently associated with outcome. A cutoff value of 5.72% of annexin V+7-AAD in monocytes on presentation is associated with poor outcome in acute TBI patients.

### 4.1. Apoptosis in TBI Patients

Following TBI, the injured brain undergoes a cascade of secondary events that include necrosis, apoptosis, and neurogenesis. Among the pathological responses that occur following TBI, apoptosis plays an important contributing role to secondary insults that lead to neuronal loss. Developmental apoptosis is thought to selectively remove unviable cells in order to promote overall growth, whereas cell death after TBI may play a much more detrimental role in recovery [24, 25]. Calcium dysregulation, excitotoxicity, activation of cysteine proteases, mitochondrial permeability transition, and mechanical perturbation of neuronal membranes are all mechanisms that contribute to apoptotic and/or necrotic neuronal cell death after TBI [26, 27]. The precise mechanism that determines the fate of a particular cell type has yet to be precisely defined.

The present study has observed injury-related differences in apoptosis levels and in cell types that undergo apoptosis among leukocytes. Generally speaking, in the present study, the percentages of apoptosis in most leukocyte subsets, including neutrophils, monocytes, and lymphocytes, are significantly higher in TBI patients than in controls. Following injury, the number of apoptotic cells is significantly increased in both outcome groups, with the poor outcome group exhibiting a more marked increase. The apoptotic response in different cell types observed in patients following TBI suggests that apoptosis may play an important role in their outcomes.

### 4.2. Early Apoptosis (Annexin V)

The two best-studied mechanisms of apoptosis are the death receptor (extrinsic) and mitochondrial (intrinsic) pathways, although there is a significant cross-talk between the two [28]. Phosphatidylserine exposure is a near-universal event in apoptosis that occurs within a few hours of the apoptotic stimulus and presents an abundant target that is readily accessible on the extracellular face of the plasma membrane [29, 30]. The anticoagulant protein annexin V shows high affinity in binding to phosphatidylserine and therefore enables the use of fluorochrome-conjugated annexin V as a marker of early stage apoptosis [31–33].

In the present study, early apoptosis in all leukocyte subsets is significantly higher in the good outcome group than the control group on Day 1 but decreased slowly thereafter (Figure 1). Only early apoptosis in lymphocytes is significantly higher in the poor outcome group on Day 1. The neutrophils and monocytes are lower in the poor outcome group than the controls, although not statistically significant. Based on these results, early apoptosis increased early may provide better outcome, which may be due to increased neural protection in early TBI.

### 4.3. APO2.7

APO2.7 is expressed on the mitochondrial membrane during early apoptosis in relation to the release of cytochrome *c* and represents activation-induced cell death under certain circumstances [34, 35]. In the present study, APO2.7 in all leukocyte subsets was significantly higher in both good and poor outcome TBI groups than in the controls on Day 1. Most of these levels persisted until Day 7 (Figure 2). However, there is no statistically significant difference in APO2.7 between good and poor outcome groups following TBI. Thus, this study concludes that activation through the mitochondria-dependent pathway in all leukocyte subsets is prominent in patients with TBI.

### 4.4. Late Apoptosis (Annexin V+7-AAD)

Annexin V+7-amino-actinomycin staining (7-AAD) is a convenient way to discriminate among early apoptosis, late apoptosis, and necrosis. Early apoptotic cells express phosphatidylserines on the outer leaflet of the plasma membrane. Phosphatidylserines can be stained by labeled annexin V. Late apoptotic and necrotic cells lose their cell membrane integrity and are permeable to vital dyes such as 7-AAD (DNA intercalator) [36].

Yakovlev et al. have shown that the gene expression for both caspase-1 and caspase-3 is increased in injured cortex at 24 h after brain injury, implicating these proteases in neuronal apoptosis induced by TBI [37]. Interestingly, the increase in caspase-3 expression in the injured cortex is temporally correlated with the peak of apoptotic cells observed in the cortex in another experimental brain injury study [19]. These data suggest that the posttraumatic altered expression of specific genes may stimulate a programmed cell death cascade resulting in apoptosis. Similar to findings of experimental brain injury studies, the poor outcome patients group in the present study has significantly elevated late apoptosis on admission compared to the control and good outcome groups. Furthermore, these increases occur in lymphocytes and monocytes, and the levels decrease dramatically on Days 4 and 7 (Figure 3). Thus, elevated late apoptosis may be used to predict the outcome.

The current study has several limitations. First, there is a relatively small sample size and a significant number of analyses performed, which may cause potential bias in statistical analysis or casual statistical finding. Second, there are many assays to evaluate for apoptosis* in vitro*, so the results and interpretations from various methodologies may be partially different. The level of apoptosis tested by flow cytometry may not necessarily reflect the real leukocyte physiologic function* in vivo*. Large-scale prospective studies are warranted to evaluate the prognostic contribution of apoptosis on clinical outcomes.

## 5. Conclusions

This study reveals the varying degrees of apoptosis between healthy individuals and patients following TBI. Leukocyte apoptosis is significantly higher in TBI patients and correlates well with outcome following injury. Late apoptosis (annexin V+7-AAD) in monocytes on presentation is also independently associated with outcome. Such differential expressions of apoptosis suggest that apoptosis in different leukocyte subsets may play an important role in the outcome following injury. Nonetheless, additional studies with a bigger study population should be performed to further corroborate these findings.

## Figures and Tables

**Table 1:** Demographic data of patients and controls on admission.

Parameter	TBI patients (*n* = 88)	Controls (*n* = 27)	*P* value
Age (y), median (IQR)	36 (20, 54)	47 (36, 59)	0.003
Male	55	13	0.263
Underlying diseases			
Hypertension	8	0	1.000
Diabetes mellitus	3	0	1.000
Alcoholism	9	0	1.000
Smoking	8	0	1.000
Clinical features at presentation			
Posttraumatic amnesia	18	NA	
Brief unconsciousness	51	NA	
Motor deficits	17	NA	
GCS at presentation	15 (13, 15)	NA	
Severity of traumatic brain injury			
Mild	68	NA	
Moderate	8	NA	
Severe	12	NA	
Injury severity score at presentation	16 (11, 20)	NA	
Laboratory data at presentation, Median (IQR)			
WBC (×10^3^/mL)	12.9 (10.4, 16.3)	5.5 (4.4, 6)	≤*0.001 *
Platelet counts (×10^3^/mL)	209 (157, 276)	211 (180, 247)	0.805
Brain imaging findings at presentation			
Depressed skull fracture	2	NA	
Pneumocranium	9	NA	
Traumatic SAH	45	NA	
Subdural hemorrhage	34	NA	
Epidural hemorrhage	16	NA	
Parenchymal contusion hemorrhage	20	NA	
Neurosurgical intervention	18	NA	
Total leukocyte apoptosis (%)			
Annexin V (%)	15.14 (10.78, 19.35)	10.86 (8.76, 13.42)	≤0.001
APO2.7 (%)	1.61 (0.99, 2.64)	0.72 (0.52, 0.85)	≤0.001
Annexin V+7-AAD (%)	3.54 (2.20, 5.59)	3.22 (2.55, 4.52)	0.600
Neutrophil apoptosis (%)			
Annexin V (%)	18.99 (12.60, 26.51)	13.9 (10.25, 17.97)	0.010
APO2.7 (%)	0.94 (0.55, 1.74)	0.51 (0.4, 0.61)	≤0.001
Annexin V+7-AAD (%)	2.33 (1.09, 5.46)	3.9 (2.23, 5.53)	0.043
Lymphocyte apoptosis (%)			
Annexin V (%)	6.31 (4.98, 8.92)	4.8 (3.82, 6.72)	0.003
APO2.7 (%)	0.78 (0.5, 1.19)	0.31 (0.22, 0.43)	≤0.001
Annexin V+7-AAD (%)	1.5 (1.13, 2.38)	1.53 (0.92, 2.09)	0.526
Monocyte apoptosis (%)			
Annexin V (%)	19.60 (15.4, 26.42)	17.38 (12.4, 21.57)	0.074
APO2.7 (%)	4.29 (1.93, 8.29)	1.75 (1.06, 2.66)	≤0.001
Annexin V+7-AAD (%)	5.0 (3.37, 8.0)	4.55 (3.27, 6.02)	0.292

CI: confidence interval; GCS: Glasgow Outcome Scale; IQR: interquartile range; 7-AAD: 7-amino-actinomycin D.

Data are presented either as absolute numbers or as medians with the interquartile range. Statistical significance was set at a level of *P* = 0.05. Statistical variance between groups was assessed by Fisher's exact test for discrete variables and by the Mann-Whitney *U* test for continuous variables.

**Table 2:** Prognostic factors of patients with acute traumatic brain injury.

	Good outcome (*N* = 79)	Poor outcome (*N* = 9)	*P* value	Odds Ratio	95% CI (lower, upper)
Age (y), median (IQR)	35 (21, 54)	48 (19, 52.5)	0.978		
Male	49	6	1.000	1.224	0.285, 5.265
Underlying diseases					
Hypertension	8	0	1.000	NA	NA
Diabetes mellitus	3	0	1.000	NA	NA
Alcoholism	8	1	1.000	1.109	0.122, 10.048
Smoking	7	1	1.000	1.285	0.139, 11.825
Clinical feature at presentation					
Posttraumatic amnesia	16	2	1.000	1.125	0.213, 5.943
Brief unconsciousness	42	9	0.009	NA	NA
Motor deficits	9	8	≤0.001	62.222	6.952, 556.89
GCS at presentation, median (IQR)	15 (14, 15)	7 (5.5, 10)	≤0.001		
Severity of traumatic brain injury			≤0.001		
Mild	67	1			
Moderate	7	1			
Severe	5	7			
Injury Severity Score at presentation, median (IQR)	16 (11, 18)	24 (16.5, 26)	0.001		
Laboratory data at presentation, Median (IQR)					
WBC (×10^3^/mL)	12.5 (10.3, 15.9)	19 (13.9, 22.4)	0.015		
Platelet counts (×10^3^/mL)	209 (157, 274)	237 (112, 348)	0.416		
Brain images findings at presentation					
Depressed skull fracture	1	1	0.195	9.75	0.555, 171.226
Pneumocranium	7	2	0.229	2.939	0.509, 16.956
Traumatic SAH	40	5	1.000	1.219	0.304, 4.877
Subdural hemorrhage	26	8	0.002	16.307	1.935, 137.387
Epidural hemorrhage	14	2	0.665	1.326	0.248, 7.076
Parenchymal contusion hemorrhage	18	2	0.97	0.968	0.184, 5.077
Neurosurgical intervention	10	8	≤0.001	55.2	6.226, 489.331
Total Leukocyte apoptosis (%)					
Annexin V (%)	15.58 (10.98, 19.35)	12.86 (9.91, 20.65)	0.407		
APO2.7 (%)	1.61 (0.97, 2.66)	1.66 (1.17, 2.50)	0.960		
Annexin V+7-AAD (%)	3.48 (2.2, 5.55)	5.76 (2.04, 8.02)	0.282		
Neutrophil apoptosis (%)					
Annexin V (%)	19.22 (14.95, 26.51)	11.27 (8.24, 25.73)	0.068		
APO2.7 (%)	0.97 (0.55, 1.97)	0.6 (0.29, 1.21)	0.154		
Annexin V+7-AAD (%)	2.48 (1.09, 6.15)	1.69 (0.91, 2.91)	0.160		
Lymphocyte apoptosis (%)					
Annexin V (%)	6.26 (4.85, 8.85)	8.19 (5.72, 14.4)	0.136		
APO2.7 (%)	0.74 (0.44, 1.17)	1.35 (0.76, 3.41)	0.058		
Annexin V+7-AAD (%)	1.44 (1.11, 2.17)	4.81 (1.84, 6.17)	0.004		
Monocyte apoptosis (%)					
Annexin V (%)	20.52 (15.93, 26.75)	13.67 (10.89, 22.66)	0.091		
APO2.7 (%)	4.16 (1.94, 7.46)	8.71 (1.70, 21.89)	0.419		
Annexin V+7-AAD (%)	4.79 (3.32, 7.12)	11.94 (4.12, 21.89)	0.022		
Acute neurosurgical complications					
Newly onset of neurological deficit	3	1	0.356	3.166	0.293, 34.131
Deterioration of consciousness	5	2	0.149	4.228	0.689, 25.935
Posttraumatic seizure	6	3	0.046	6.083	1.207, 30.637
Days of ICU stay	2 (0, 3)	11 (8.5, 16.5)	≤0.001		
Days of hospitalization	9 (7, 13)	17.5 (11, 24.25)	0.001		

CI: confidence interval; GCS: Glasgow Outcome Scale; IQR: interquartile range; 7-AAD: 7-amino-actinomycin D.

Data are presented either as absolute numbers or as medians with the interquartile range. Statistical significance was set at a level of *P* = 0.05. Statistical variance between groups was assessed by Fisher's exact test for discrete variables and by the Mann-Whitney *U* test for continuous variables.
